# Cultivation strategies for production of (*R*)-3-hydroxybutyric acid from simultaneous consumption of glucose, xylose and arabinose by *Escherichia coli*

**DOI:** 10.1186/s12934-015-0236-2

**Published:** 2015-04-11

**Authors:** Johan Jarmander, Jaroslav Belotserkovsky, Gustav Sjöberg, Mónica Guevara-Martínez, Mariel Pérez-Zabaleta, Jorge Quillaguamán, Gen Larsson

**Affiliations:** School of Biotechnology, Division of Industrial Biotechnology, KTH Royal Institute of Technology, SE 106 91 Stockholm, Sweden; Center of Biotechnology, Faculty of Science and Technology, Universidad Mayor de San Simón, Cochabamba, Bolivia

**Keywords:** *Escherichia coli*, 3-Hydroxybutyric acid, 3HB, Simultaneous uptake, Lignocellulose, Production process, Nitrogen limitation

## Abstract

**Background:**

Lignocellulosic waste is a desirable biomass for use in second generation biorefineries. Up to 40% of its sugar content consist of pentoses, which organisms either take up sequentially after glucose depletion, or not at all. A previously described *Escherichia coli* strain, PPA652ara, capable of simultaneous consumption of glucose, xylose and arabinose was in the present work utilized for production of (*R*)-3-hydroxybutyric acid (3HB) from a mixture of glucose, xylose and arabinose.

**Results:**

The *Halomonas boliviensis* genes for 3HB production were for the first time cloned into *E. coli* PPA652ara, leading to product secretion directly into the medium. Process design was based on comparisons of batch, fed-batch and continuous cultivation, where both excess and limitation of the carbon mixture was studied. Carbon limitation resulted in low specific productivity of 3HB (<2 mg g^−1^ h^−1^) compared to carbon excess (25 mg g^−1^ h^−1^), but the yield of 3HB/cell dry weight (Y_3HB/CDW_) was very low (0.06 g g^−1^) during excess. Nitrogen-exhausted conditions could be used to sustain a high specific productivity (31 mg g^−1^ h^−1^) and to increase the yield of 3HB/cell dry weight to 1.38 g g^−1^. Nitrogen-limited fed-batch process design led to further increased specific productivity (38 mg g^−1^ h^−1^) but also to additional cell growth (Y_3HB/CDW_ = 0.16 g g^−1^). Strain PPA652ara did under all processing conditions simultaneously consume glucose, xylose and arabinose, which was not the case for a reference wild type *E. coli*, which also gave a higher carbon flux to acetic acid.

**Conclusions:**

It was demonstrated that by using *E. coli* PPA652ara, it was possible to design a production process for 3HB from a mixture of glucose, xylose and arabinose where all sugars were consumed. An industrial 3HB production process is proposed to be divided into a growth and a production phase, and nitrogen depletion/limitation is a potential strategy to maximize the yield of 3HB/CDW in the latter. The specific productivity of 3HB reported here from glucose, xylose and arabinose by *E. coli* is further comparable to the current state of the art for production from glucose sources.

## Background

Lignocellulosic waste is an abundantly available, low cost and generally unutilized biomass source that can contain up to 70–80% (w/w) carbohydrates [1]. This makes it desirable as a substrate for the various chemicals proposed for production in second generation biorefineries. The relative sugar composition of for example wheat straw after dilute acid pretreatment and enzymatic saccharification is 55.6% (D-)glucose, 37.6% (D-)xylose and 4.1% (L-)arabinose (w/w) [2]. Approximately 40% of the total sugar content is thus composed of pentoses, which current production organisms cannot consume efficiently. For example, wild type *Saccharomyces cerevisiae* can neither metabolize xylose nor arabinose [3]. Without efficient conversion of the pentoses into biomass and product, the carbon source is in effect only utilized to ~ 60% of its potential. There have been efforts to develop *S. cerevisiae* strains able to co-ferment glucose and xylose, but the uptake has been either slow or sequential, which leads to residual sugar in the medium [4,5]. In *Escherichia coli*, on the other hand, it was previously reported [6] that the ability to absorb pentoses is strain dependent and subjected to a complex sequential uptake chain that is initiated only at low glucose levels in the cultivation medium. In *E. coli*, the three most abundant sugars of pretreated wheat straw are thus taken up in the order of glucose > arabinose > xylose. This is due to a combination of carbon catabolite repression [7], inducer exclusion and pentose uptake regulation [8].

In a previous study, an *E. coli* strain (named PPA652ara) capable of simultaneous metabolism of glucose, xylose and arabinose under aerobic conditions without leaving any residual sugar in the cultivation medium was designed [6]. This was achieved by deletion of the *ptsG* gene (EIICB^Glc^), the preferred inner membrane transporter for glucose. This deletion leads to inactivation of the regulatory mechanism of inducer exclusion, but also to increased production of cAMP, the activator of most sugar metabolism operons. Binding of cAMP to these operons should thus lead to continuous promoter transcription and expression of the respective permeases, thereby abolishing also the catabolite repression control. Pentose uptake in *E. coli* is regulated by arabinose repression of xylose uptake, and by using a technique of precultivation on arabinose, this regulatory layer could also be removed in PPA652ara.

The strain PPA652ara was intended to be a platform for efficient aerobic production processes of chemicals from lignocellulosic waste. The current work therefore investigates the effect of recombinant product formation on the utilization of mixed sugars by this strain. As such, PPA652ara was used to produce the hydroxyalkanoic acid (*R*)-3-hydroxybutyric acid (abbreviated 3HB from now on). 3HB is the monomeric unit of poly-(*R*)-3-hydroxybutyric acid (PHB), a biodegradable polyester that is naturally produced by several microorganisms, however not by *E. coli* [9]. PHB metabolism is initiated from a condensation of two acetyl-CoA molecules to form acetoacetyl-CoA, catalyzed by the enzyme acetoacetyl-CoA thiolase [10]. Acetoacetyl-CoA is then stereoselectively reduced to (R) or (S)-3HB-CoA by a NAD(P)H dependent acetoacetyl-CoA reductase. PHB synthase lastly catalyzes the polymerization of the monomer to PHB.

The 3HB monomer is an interesting molecule since it contains a chiral center and two active functional groups that can be easily modified: a hydroxyl group and a carboxyl group [11,12]. This allows it to be used as building block in the synthesis of several chiral fine chemicals, pharmaceuticals and copolyesters. 3HB has been produced by both chemical synthesis [13] and biotechnological processes [12-15]. The biotechnological processes have so far been the most promising due to the advantage of producing an enantiomerically pure product, and the ability to do so without the use of organic solvents. 3HB has been produced in *E. coli* by expression of genes from wild type PHB producers, predominantly *Cupriavidus necator* (previously known as *Ralstonia eutropha* and *Alcaligenes eutrophus*) by various strategies [16-19]. In these studies, titers of 3HB between 7.3-12.2 g L^−1^ were achieved from glucose as the carbon source in 24–96 h of cultivation, having volumetric productivities in the range of 0.07-0.50 g L^−1^ h^−1^.

In the current work, the aim was to use the platform *E. coli* PPA652ara to design a production process of 3HB from a mixture of glucose, xylose and arabinose, a typical composition in waste biomass, in which all monosaccharides are consumed. The genes for 3HB production were cloned from the halophilic, naturally PHB-producing bacterium *Halomonas boliviensis*, which has been shown to be a promising producer of this chemical [20]. To our knowledge, this is the first time that genes from *H. boliviensis* have been used for recombinant production of 3HB in *E. coli*. After 3HB production had been verified, the best strategy to enhance 3HB productivity was sought. This was based on different cultivation concepts using nutrient excess in combination with exhaust or limitation strategies.

## Results and discussion

### Validation of 3HB metabolism in *E. coli* from mixed sugars

**Figure 1:**
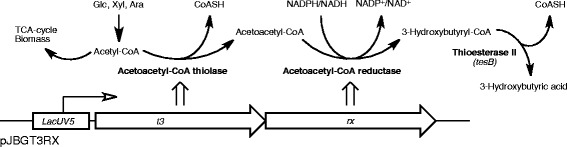
**3HB pathway introduced into** ***E. coli*** **by plasmid pJBG.** Genes *t3* (acetoacetyl-CoA thiolase) and *rx* (acetoacetyl-CoA reductase) were cloned from *H. boliviensis* into plasmid pJBG (pACYC184-derived) to produce pJBGT3RX (shown in figure by the gene order *t3*-*rx*) and pJBGRXT3 (gene order *rx*-*t3*). The plasmids were subsequently transformed into *E. coli* AF1000 and PPA652ara and used to produce 3HB in the medium. Conversion of 3HB-CoA into 3HB is likely catalyzed by the *E. coli* native enzyme thioesterase II (*TesB*).

To produce 3HB recombinantly, the genes *t3* and *rx*, encoding acetoacetyl-CoA thiolase and acetoacetyl-CoA reductase respectively, were cloned from *H. boliviensis* [20] into the low-copy-number plasmid pJBG. Two plasmids were constructed: pJBGT3RX (gene order *t3*-*rx*) and pJBGRXT3 (*rx*-*t3*), where gene expression was controlled by the LacUV5 promoter. The plasmids were transformed into the *E. coli* PPA652ara platform as well as the wild type parental strain AF1000 [21], used as a reference. The 3HB pathway introduced into the strains by the plasmids is illustrated in Figure 1.

AF1000 with pJBGT3RX or pJBGRXT3 was first cultivated on glucose minimal medium in shake flasks. From this, it could be verified that 3HB was successfully produced and secreted to the medium from both constructs when induced with isopropyl β-D-1-thiogalactopyranoside (IPTG) to a concentration of 200 μM, and thus that the gene order did not seem to have a significant effect (pJBGT3RX: 0.19 g L^−1^, pJBGRXT3: 0.18 g L^−1^, Table 1). Without induction, only trace amounts of 3HB were secreted. There was no accumulation of 3HB in the cell pellet as the measured amount was below the detection limit of the analysis assay (<0.004 g L^−1^). By gas chromatography, it was subsequently confirmed that only the R-enantiomer of 3HB was produced. Since 3HB was present in the medium, this suggested that the 3HB-CoA intermediate produced by acetoacetyl-CoA reductase (*rx*) freely degrades into 3HB and CoASH in the present *E. coli* background. The responsible enzyme is likely to be an unspecific thioesterase, such as *tesB* (as illustrated in Figure 1), which in a previous study has been overexpressed to increase 3HB production in *E. coli* [17]. As a control, potential PHB synthesis was also analyzed and was neither detected in the medium nor in the cell pellet.

**Table 1:** **3HB production by assorted** ***E. coli*** **strain and plasmid combinations on varied sugar carbon sources**

***E. coli*** **strain**	**Plasmid**	**Carbon source**	**IPTG**	**Growth rate**	**Final OD**_**600**_	**3HB**	**Y**_**3HB/OD**_
			**(μM)**	**(h** ^**−1**^ **)**		**(g L** ^**−1**^ **)**	**(g L** ^**−1**^ **OD**_**600**_^**−1**^ **)**
AF1000	pJBGT3RX	Glc	/	0.74	3.5	0.00	/
AF1000	pJBGT3RX	Glc	200	0.74	3.0	0.19	0.06
AF1000	pJBGRXT3	Glc	/	0.80	3.6	0.00	/
AF1000	pJBGRXT3	Glc	200	0.78	3.4	0.18	0.05
PPA652ara	pJBGT3RX	Glc	200	0.35	3.4	0.01	/
PPA652ara	pJBGT3RX	Xyl	200	0.46	4.4	0.18	0.04
PPA652ara	pJBGT3RX	Ara	200	0.45	3.2	0.17	0.05
PPA652ara	pJBGT3RX	Glc + Xyl + Ara	200	0.56	3.2	0.17	0.05

OD_600_ = Optical density at 600 nm.

For the multiple sugar based process design, pJBGT3RX was chosen, and used in the platform strain. This construct was initially cultivated on glucose, xylose and arabinose individually, but also as a mixture (Table 1). When cultivated on xylose or arabinose, the amount of 3HB from PPA652ara (xylose: 0.18 g L^−1^, arabinose: 0.17 g L^−1^) was not significantly different from that of the wild type AF1000 cultivated on glucose, confirming that 3HB can be produced from these pentoses with similar efficiency. However, when glucose was used as the single carbon source for PPA652ara, the concentration of secreted 3HB was practically zero. This could possibly be an effect related to the lower specific uptake rate of glucose of PPA652ara, which lacks the primary glucose inner membrane transporter, *ptsG*, and thus has approximately twice the doubling time of AF1000 (118 min vs. 56 min) on glucose. Nevertheless, the substrate that ultimately will be used is a carbohydrate mixture, and when PPA652ara was cultivated on glucose, xylose and arabinose, it produced 3HB to levels (0.17 g L^−1^) that were comparable to AF1000 cultivated on glucose.

### Carbon-limited process design

**Figure 2:**
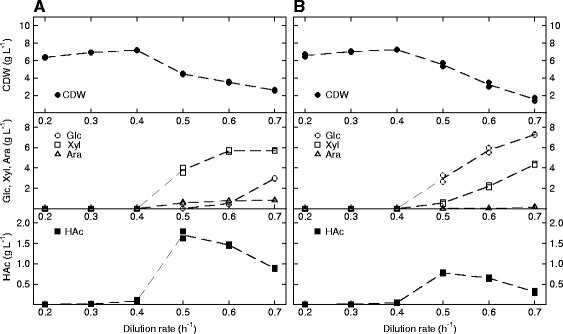
**Growth of** ***E. coli*** **AF1000 (A) and PPA652ara (B) on glucose, xylose and arabinose in continuous mode without product formation.** Parameters: cell dry weight (CDW, filled circles), glucose (Glc, open circles), xylose (Xyl, open squares), arabinose (Ara, open triangles) and acetic acid (HAc, filled squares). Each dilution rate was tested in duplicate and the mean values of the two sample series are represented as dashed lines.

To increase the volumetric productivity, biotechnological production processes of industrial scale are most often run in fed-batch or continuous cultivation mode with a desired limiting component, usually the carbon source. Feeding of a mixture of glucose, xylose and arabinose can be proposed to lead to accumulation of the pentoses since these sugars are consumed sequentially by wild type *E. coli* when available in excess. It is thus imperative to determine the feeding regime in which this accumulation is initiated so that the limitation can be upheld. This was evaluated in continuous mode at a dilution rate (D) varying between 0.2 h^−1^ and 0.7 h^−1^, initially without the 3HB production plasmid (Figure 2). The following sugar concentrations were used: 8.20 g L^−1^ glucose, 5.95 g L^−1^ xylose and 0.85 g L^−1^ arabinose (15.0 g L^−1^ in total), equivalent to the relative ratios of a pretreated wheat straw hydrolyzate [2]. In the case of AF1000 (Figure 2A), which has an active regulation of sugar uptake, it was quite unexpected to see that up to a dilution rate of 0.4 h^−1^, there was no accumulation of pentoses, indicating that the glucose concentration under these conditions were not high enough to trigger catabolite repression. First at a dilution rate of 0.5 h^−1^ and higher, the parental strain entered the feeding regime where xylose and arabinose accumulated, resulting in a declining cell dry weight (CDW). This means that a carbon-limited fed-batch or continuous cultivation could most likely be run up to a rate of 0.4 h^−1^ for AF1000. Interestingly, this was similar for the platform strain PPA652ara (Figure 2B). Here it is however mainly glucose that starts to accumulate at D = 0.5 h^−1^, likely explained by the fact that the maximum glucose uptake rate is lower for the mutant strain than for the wild type. As the dilution rate was increased further, the concentration of xylose also increased in the reactor.

Byproduct formation for these two strains was however considerably different, and the acetic acid concentrations for AF1000 were over 200% of the values for PPA652ara (peaking at 1.79 g L^−1^ vs. 0.78 g L^−1^ at dilution rate 0.5 h^−1^). This is not desirable and will be a drawback for the use of the parental strain. As catabolite repression was found to require relatively high glucose fluxes, the conclusion from carbon-limited growth is that both strains can effectively run at the same maximum feed rate for simultaneous sugar uptake and the reduced acetic acid accumulation appears to be the only explicit benefit in using PPA652ara over a wild type *E. coli* in sugar-limited processes.

**Figure 3:**
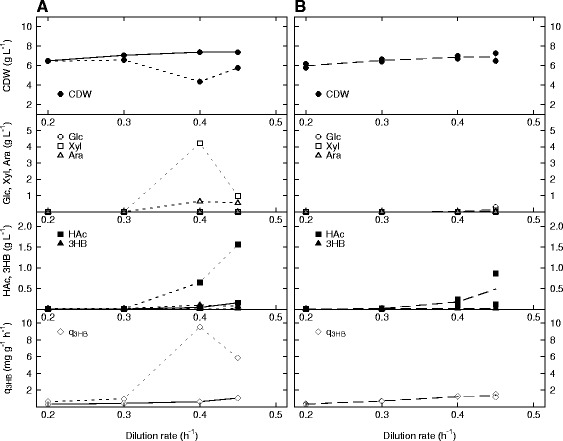
**Production of 3HB in** ***E. coli*** **AF1000 (A) and PPA652ara (B) during cultivation on glucose, xylose and arabinose in continuous mode.** Parameters: cell dry weight (CDW, filled circles), glucose (Glc, open circles), xylose (Xyl, open squares), arabinose (Ara, open triangles), acetic acid (HAc, filled squares), 3HB (filled triangles) and specific productivity of 3HB (q_3HB_, open diamonds). Each dilution rate was tested in duplicate. In A, the dotted and solid lines each represent one sample series. In B, the mean values of the two sample series are represented as dashed lines.

This experiment was repeated with the difference that AF1000 and PPA652ara were now harboring pJBGT3RX (Figure 3) and the medium was supplemented with IPTG. AF1000 (Figure 3A) did under these conditions appear to be unstable at first, since xylose and arabinose were accumulating already at a dilution rate of 0.4 h^−1^ in the first cycle (dotted line), just to be consumed again when the dilution rate was further increased to 0.45 h^−1^. When the same dilution rates were tested again in a second cycle (solid line), this behavior was not repeated and was in agreement with the results of Figure 2A. It is thus possible that AF1000 that produces 3HB needs some time to adapt to mixed carbon uptake. PPA652ara with pJBGT3RX (Figure 3B) seemed to be a more stable construct, as the sugar accumulation pattern resembled that when no plasmid was used (Figure 2B), and there was no major difference between two sample series. As can be seen in Figure 3, 3HB concentrations, and subsequently also the specific productivity of 3HB (q_3HB_) were very low for both strains. Generally, levels of 0.01-0.02 g L^−1^ were reached except at D = 0.4 h^−1^ for AF1000, which gave ~ 0.10 g L^−1^, resulting in a specific productivity of ~10 mg g^−1^ h^−1^.

### Process design with excess of carbon

**Figure 4:**
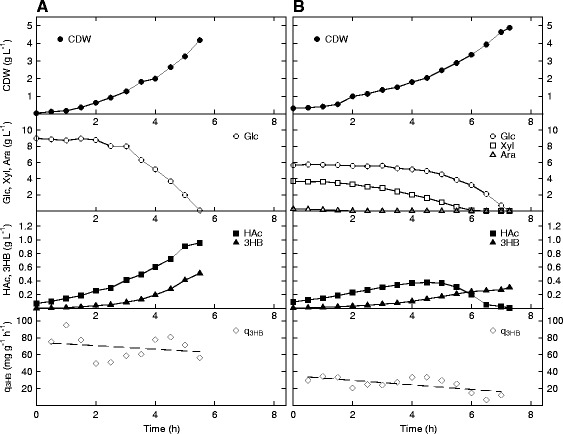
**Production of 3HB in** ***E. coli*** **AF1000 (A) and PPA652ara (B) during cultivation on glucose (AF1000), or glucose, xylose and arabinose (PPA652ara) in batch mode.** Parameters: cell dry weight (CDW, filled circles), glucose (Glc, open circles), xylose (Xyl, open squares), arabinose (Ara, open triangles), acetic acid (HAc, filled squares), 3HB (filled triangles) and specific productivity of 3HB (q_3HB_, open diamonds). The specific productivity of 3HB has been curve fitted to a 1st order polynomial.

Natural PHB-producing organisms, such as *H. boliviensis* and *C. necator,* synthesize the polyester as a carbon storage compound during excess of carbon and deficiency of other nutrients [10]. It seems likely that the carbon flux in *E. coli* could, by a similar strategy, be forced to the product through condensation of two moles of acetyl-CoA, avoiding the tricarboxylic acid (TCA) cycle and subsequent biomass formation by citrate synthase inhibition. As a first step, 3HB-production at high sugar concentrations was evaluated. Both AF1000 (Figure 4A) and PPA652ara (Figure 4B) with pJBGT3RX were therefore cultivated in batch mode in bioreactors, AF1000 on 10.00 g L^−1^ glucose, and PPA652ara on 5.47 g L^−1^ glucose, 3.97 g L^−1^ xylose and 0.56 g L^−1^ arabinose (10.00 g L^−1^ in total). The cultivation of AF1000 on the sugar mixture is not shown here as these sugars will be taken up in a sequential fashion that has been shown before [6]. As seen in the figure, this was clearly not the case with PPA652ara that consumes all available sugar simultaneously. However, as expected from a higher growth rate (μ, 0.76 h^−1^ vs. 0.39 h^−1^), AF1000 consumed the glucose in approximately 75% of the time needed for PPA652ara to take up the sugar mixture.

In correlation with the results of Figure 3, PPA652ara produces significantly less of the by-product acetic acid than AF1000, although a high overflow metabolism at sugar excess is expected for both strains. For the platform strain, the acetic acid is also completely reabsorbed. AF1000 did have a higher q_3HB_ than PPA652ara (on average 68 mg g^−1^ h^−1^ vs. 25 mg g^−1^ h^−1^), which was reflected in the higher final titer of 3HB at 0.51 g L^−1^, compared to 0.31 g L^−1^ for PPA652ara. Also, the specific uptake rate of sugar (q_S_) was higher for AF1000 solely on glucose than the q_S total_ (the combined specific uptake rate of glucose, xylose and arabinose) for PPA652ara (on average 1.00 g g^−1^ h^−1^ vs. 0.70 g g^−1^ h^−1^). From this experiment, it is clear that a non-limited sugar flux is necessary for high specific productivity of 3HB, as both AF1000 and PPA652ara display q_3HB_ values that are several magnitudes greater than in sugar-limited continuous mode.

### Process design with excess of carbon and nitrogen exhaustion

**Figure 5:**
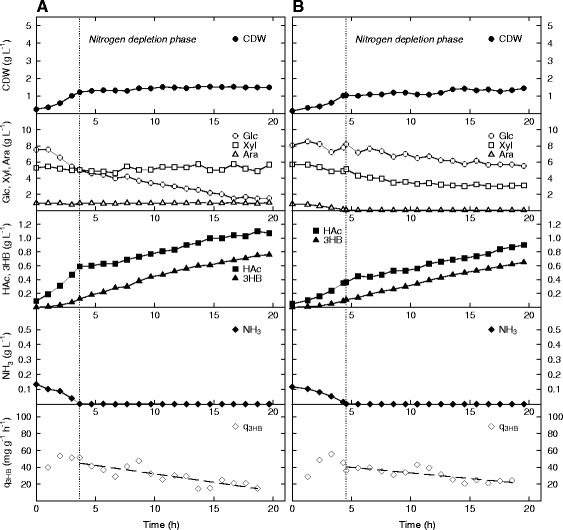
**Production of 3HB in** ***E. coli*** **AF1000 (A) and PPA652ara (B) during cultivation on glucose, xylose and arabinose in batch mode during nitrogen depletion.** Parameters: cell dry weight (CDW, filled circles), glucose (Glc, open circles), xylose (Xyl, open squares), arabinose (Ara, open triangles), acetic acid (HAc, filled squares), 3HB (filled triangles), ammonia (NH_3_, filled diamonds) and specific productivity of 3HB (q_3HB_, open diamonds). The specific productivity of 3HB in the nitrogen depletion phase has been curve fitted to a 1st order polynomial.

A fully optimized production process of 3HB with high volumetric productivity is likely to be divided into (at least) two phases: the first being a cell growth phase, and the latter a 3HB production phase. In the 3HB production phase, it will be essential to minimize cell growth and byproduct formation so that a larger quota of the available carbon can be directed towards the product. It had already been established that an excess of carbon was crucial for high specific productivity of 3HB, but the question was if this level of productivity could be maintained without substantially increasing the biomass further. It was therefore decided to investigate the specific productivity of 3HB during nitrogen depletion while sugars were aplenty. AF1000 and PPA652ara with pJBGT3RX were consequently cultivated on the same sugar mixture as before but the minimal salt medium was designed to be depleted of the single nitrogen source, ammonia (NH_3_), after four hours (Figure 5). For both strains, nitrogen became depleted at a CDW of slightly more than 1 g L^−1^, but as can be seen in the figure, both AF1000 (Figure 5A) and PPA652ara (Figure 5B) continued to take up sugar also in the nitrogen-depleted phase (the start of this phase is indicated by the dotted lines in the figure). As previously observed [6], AF1000 did however only take up the glucose quota of the sugar mixture while PPA652ara consumed all three sugar sources throughout the cultivation.

During nitrogen depletion, production of 3HB continued for both strains at a steady rate, with titers of 0.64 g L^−1^ for AF1000, and 0.54 g L^−1^ for PPA652ara in the nitrogen depletion phase. For PPA652ara, this is a distinct improvement in productivity compared to the results of the batch cultivation with excess of carbon and nitrogen (Figure 4). The mean values of q_3HB_ decayed over time for both strains in the nitrogen depletion phase, which is to be expected, as nitrogen is not refilled in the process. The two strains showed very similar mean values of q_3HB_ at 29 mg g^−1^ h^−1^ for AF1000 and 31 mg g^−1^ h^−1^ for PPA652ara. For PPA652ara, this is an improvement of 25% over the previously mentioned batch cultivation, but for AF1000, it is instead a considerable decrease of approximately 60%. As expected, the values of q_S total_ were much lower in this experiment (on average approximately 0.2 g g^−1^ h^−1^ for AF1000, and 0.3 g g^−1^ h^−1^ for PPA652ara). It is also noteworthy that acetic acid continues to be produced in the nitrogen depletion phase, with PPA652ara producing the most (0.48 g L^−1^ for AF1000 vs. 0.54 g L^−1^ for PPA652ara).

### Nitrogen limited fed-batch cultivation

The experiment performed in Figure 5 allowed us to deduce that it is possible to maintain a high specific productivity of 3HB from a mixture of glucose, xylose and arabinose without increasing the cell mass substantially by depleting nitrogen. In order to avoid a decaying production rate of 3HB, it is likely that nitrogen needs to be replenished. It is however probably not desirable to use a high feeding rate, as this allows for more of the carbon to be directed towards cell mass, rather than 3HB.

**Figure 6:**
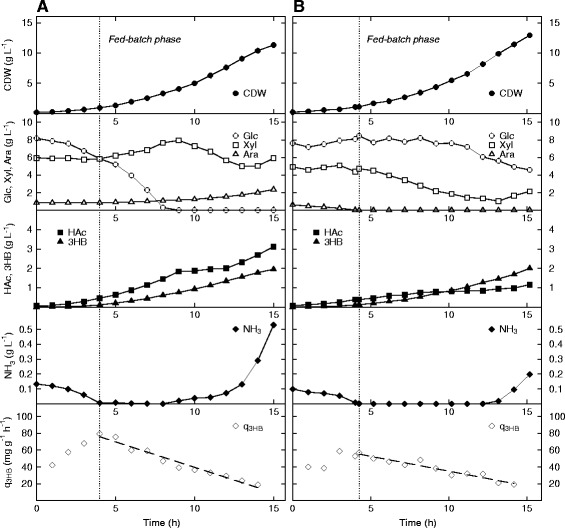
**Production of 3HB in** ***E. coli*** **AF1000 (A) and PPA652ara (B) during cultivation on glucose, xylose and arabinose in nitrogen-limited fed-batch mode.** Parameters: cell dry weight (CDW, filled circles), glucose (Glc, open circles), xylose (Xyl, open squares), arabinose (Ara, open triangles), acetic acid (HAc, filled squares), 3HB (filled triangles), ammonia (NH_3_, filled diamonds) and specific productivity of 3HB (q_3HB_, open diamonds). The specific productivity of 3HB has been curve fitted to a 1st order polynomial.

With these considerations in mind, AF1000 and PPA652ara with pJBGT3RX were cultivated in nitrogen-limited fed-batch mode, starting from a batch culture with 1.00 g L^−1^ ammonium sulfate (equivalent to 0.128 g L^−1^ ammonia), as can be seen in Figure 6. Once nitrogen became depleted in the reactors, ammonium sulfate, and also sugars (in the same ratio as in the starting batch) were fed exponentially to keep a growth rate of 0.2 h^−1^ (The start of the fed-batch phase is indicated by the dotted line in the figure). The feeding rate was chosen arbitrarily to replenish nitrogen, but to not give a substantial increase in biomass formation. As before, only glucose was initially consumed by AF1000 (Figure 6A). Since the sugars were being continually fed into the reactor, this led to that xylose and arabinose accumulated to higher than the initial concentrations. Glucose levels became very low approximately midway through the experiment, at which time xylose, but not arabinose, started to be consumed. Due to the initial accumulation, the final xylose concentration was close to the starting value. PPA652ara on the other hand consumed all three sugars continuously (Figure 6B), as expected from the previous cultivations. Ammonia accumulated in both processes, but at considerably different times. For AF1000 this took place in the middle of the process once glucose was almost in exhaust, and a final concentration of 3.88 g L^−1^ was registered. For PPA652ara this only took place in the very end, and to a final concentration of 1.45 g L^−1^.

For both strains, an average growth rate of 0.24 h^−1^ was achieved in the nitrogen limited fed-batch phase, which was slightly higher than expected. The average q_S total_ values were in turn also approximately the same, at 0.6 g g^−1^ h^−1^. Even the 3HB titers attained were very similar for the two strains, at 1.84 and 1.87 g L^−1^ respectively for AF1000 and PPA652ara. The acetic acid concentration was however much higher for AF1000 at 2.66 g L^−1^, to compare with 0.77 g L^−1^ for PPA652ara. The specific productivity of 3HB in the fed-batch phase was further higher for both strains than during nitrogen depletion (Figure 5), with average values of 45 mg g^−1^ h^−1^ (55% increase) and 38 mg g^−1^ h^−1^ (22% increase) achieved for AF1000 and PPA652ara respectively. Even though the specific productivities of 3HB increased during nitrogen-limited fed-batch, feeding nitrogen into the bioreactor did not significantly reduce their time-dependent decay. These results instead resemble what has been seen in the past for induced expression systems building on substrate induced promoters [21], which after a short time show clear saturation kinetics. The use of a constitutive promoter could possibly result in a more stable specific productivity of 3HB.

### Comparison of process modes

The results of using different process modes for production of 3HB by the platform *E. coli* PPA652ara and the wild type reference AF1000 are summarized in Table 2. It is quite evident that one would need to divide a production process of 3HB from glucose, xylose and arabinose by PPA652ara into a growth, and a production phase. This is mainly because of the low yield of 3HB/CDW (Y_3HB/CDW_) achieved in batch mode at 0.06 g g^−1^, despite a decent mean q_3HB_ of 25 mg g^−1^ h^−1^. By depleting nitrogen in the form of ammonia, the Y_3HB/CDW_ is increased considerably, going up to 1.38 g g^−1^, while the mean value of q_3HB_ is slightly increased to 31 mg g^−1^ h^−1^. Nitrogen depletion is thus an effective method for reducing biomass formation and steering the carbon flux towards 3HB in the production phase. Feeding ammonium sulfate and sugars at a rate of 0.2 h^−1^ increased the mean q_3HB_ further to 38 mg g^−1^ h^−1^, but this was not enough to compensate for the additional biomass formed, as the Y_3HB/CDW_ in fed-batch mode was 0.16 g g^−1^, approximately 8 times lower than during nitrogen depletion. When comparing PPA652ara with the reference strain AF1000, in addition to its unique ability to consume glucose, xylose and arabinose simultaneously, PPA652ara generally reaches higher biomasses than AF1000. This can probably be explained by the fact that AF1000 produces considerably more acetic acid, an effect that likely is a consequence of AF1000’s much greater specific uptake rate of glucose.

**Table 2:** **Comparison of production of 3HB by** ***E. coli*** **AF1000 and PPA652ara by varied cultivation techniques**

**Process mode**	**Sugar**	**Strain**	**Time**	**CDW**	**Sugar consumed**	**3HB**	**HAc**	**mean q**_**S total**_	**mean q**_**3HB**_	**Y**_**3HB/HAc**_	**Y**_**3HB/CDW**_	**Y**_**3HB/Sugar**_
			**(h)**	**(g L** ^**−1**^ **)**	**(g L** ^**−1**^ **)**	**(g L** ^**−1**^ **)**	**(g L** ^**−1**^ **)**	**(g g** ^**−1**^ **h** ^**−1**^ **)**	**(mg g** ^**−1**^ **h** ^**−1**^ **)**	**(g g** ^**−1**^ **)**	**(g g** ^**−1**^ **)**	**(g g** ^**−1**^ **)**
Batch	Glc	AF1000	5.5	4.18	8.90	0.51	0.96	1.0	68	0.54	0.12	0.06
Batch	Glc + Xyl + Ara	PPA652ara	7.3	4.88	9.65	0.31	0.00	0.7	25	→ ∞	0.06	0.03
N-depletion	Glc + Xyl + Ara	AF1000	16.0	0.27	2.73	0.64	0.48	0.2	29	1.33	2.37	0.23
N-depletion	Glc + Xyl + Ara	PPA652ara	15.0	0.39	3.79	0.54	0.54	0.3	31	1.00	1.38	0.14
N-fedbatch	Glc + Xyl + Ara	AF1000	11.0	10.37	37.05	1.84	2.66	0.6	45	0.69	0.18	0.05
N-fedbatch	Glc + Xyl + Ara	PPA652ara	11.0	11.82	36.75	1.87	0.77	0.6	38	2.43	0.16	0.05

In the cases of nitrogen depletion and nitrogen-limited fed-batch, the values given in the table are calculated for the respective depleted/limited phase only, and do not include the batch phases.

The fluctuating level of CoA in the cell during different cultivation conditions is one plausible explanation for the big variance seen in the Y_3HB/CDW_. This would be due to inhibition of acetoacetyl-CoA thiolase by high levels of CoA [22], which is likely during exponential growth in batch experiments. In batch mode, the majority of the carbon would thus be directed towards biomass by the competing enzyme citrate synthase in the TCA cycle. When nitrogen is depleted, the concentration of CoA is however expected to be low, and the activity of acetoacetyl-CoA thiolase should therefore increase. In addition, the intracellular concentration of NADH and/or ATP is expected to go up during nitrogen starvation, which in turn leads to inhibition of citrate synthase. The end result is increased 3HB secretion during nitrogen depletion, which is what was seen in the experiments. Since the nitrogen-limited fed-batch cultivations resulted in significantly reduced Y_3HB/CDW_ compared to nitrogen-depletion, this gives that citrate synthase likely was much more active during the fed-batch conditions. If nitrogen needs to be restocked during the 3HB production phase, it should therefore likely be fed at a rate lower than 0.2 h^−1^ in order to avoid unwanted biomass formation.

To our knowledge, the highest volumetric productivity of 3HB that has been reported for recombinant *E. coli* is 12.2 g L^−1^ achieved in 24 h from glucose, which is equal to 0.50 g L^−1^ h^−1^ [17]. To achieve the same result with PPA652ara under nitrogen depletion, the cell growth phase should be extended to approximately 16 g L^−1^ CDW. This is quite possible by use of standard *E. coli* cultivation protocols and the realistic limit of the biomass is probably much higher [23]. PPA652ara thus seems a promising candidate strain for production of chemicals from sugar mixtures of comparable productivity as the current state of the art *E. coli* from glucose.

## Conclusions

In this work, the *E. coli* platform strain PPA652ara was used to successfully produce 3HB from a mixture of glucose, xylose and arabinose, in which all sugars were being consumed. In addition, simultaneous consumption of the sugars by PPA652ara resulted in less acetic acid being produced than by the wild type strain AF1000, from a sequential sugar uptake. In order to obtain a high specific productivity of 3HB in combination with a high yield of 3HB/CDW, carbon needed to be available in excess and another essential nutrient, such as nitrogen, limited. The specific productivity of 3HB reported here by strain PPA652ara from a mixture of glucose, xylose and arabinose, is further comparable to the values achieved by *E. coli* from glucose in the current state of the art.

## Methods

### Bacterial strains and plasmids

The *E. coli* strains used in this work were AF1000 (*MC4100, relA1*^*+*^) [21] and PPA652ara (*MC4100, relA1*^*+*^*, ΔptsG::Km*, adapted to growth on L-arabinose) [6]. The 3HB production plasmids pJBGT3RX and pJBGRXT3 were constructed from pKM1D, a pACYC184-derived low copy number plasmid with *ori* p15A, a lacUV5 promoter, a multiple cloning site, the *lacIq* repressor and a chloramphenicol resistance gene. From *H. boliviensis*, the two genes needed to produce 3HB, *t3* (acetoacetyl-CoA thiolase) and *rx* (acetoacetyl-CoA reductase) were amplified by PCR with complimentary tails for cloning between the HindIII and SacI sites on pKM1D. The plasmid was digested by HindIII and SacI, and the two genes were cloned by the SLIC method [24] in two alternate gene orders, either thiolase-reductase (in pJBGT3RX) or reductase-thiolase (in pJBGRXT3).

### Cultivation procedure

#### Batch cultivation in shake flask

*E. coli* cells harboring pJBGT3RX or pJBGRXT3 were taken from a glycerol stock stored at −80°C and used to inoculate sterile baffled 1000 mL shake flasks containing 100 mL cultivation medium. The cells were cultivated at 37°C and 180 rpm shaking until the stationary phase was reached, at which time the production of 3HB was evaluated. Production of 3HB was induced by the addition of 200 μM IPTG at an optical density at 600 nm (OD_600_) of 0.2.

#### Continuous cultivation in bioreactor

*E. coli* cells harboring pJBGT3RX were taken from a glycerol stock stored at −80°C and used to inoculate sterile baffled 1000 mL shake flasks containing 100 mL cultivation medium. The cells were cultivated overnight at 37°C and 180 rpm and were used to inoculate a sterile 3 L stirred tank bioreactor (STR) containing 2 L cultivation medium. A cultivation temperature of 37°C was used in the reactor and dissolved oxygen (DO) was maintained at above 30%. Antifoam was added when required. Once the cells had consumed the sugar in the medium, the inlet feed was started and continuous mode was sustained through a weight-based efflux of cell culture. At least 4 residence times were allowed to pass before the reactor was sampled after any conditions were changed. Each dilution rate was analyzed in duplicate. For production of 3HB, IPTG was present in the cultivation medium from the start at a concentration of 200 μM.

#### Batch and fed-batch cultivation in bioreactor

*E. coli* cells harboring pJBGT3RX were taken from a glycerol stock stored at −80°C and used to inoculate a sterile 10 L STR containing 6 or 7 L cultivation medium. The cells were cultivated at 37°C and DO was maintained at above 30%. Antifoam was added when required. Production of 3HB was induced by the addition of 200 μM IPTG at an OD_600_ of 0.2. For fed-batch cultivations, feeding was initiated once ammonia was depleted. Samples for OD_600_, cell dry weight, glucose, xylose, arabinose, ammonia, 3HB and acetic acid were taken regularly during the bioreactor cultivations.

#### Cultivation medium

The cultivation medium used was a minimal salt medium consisting of (for non-nitrogen limited cultures, per liter): 5.00 g (NH_4_)_2_SO_4_, 1.60 g KH_2_PO_4_, 6.60 g Na_2_HPO_4_●2 H_2_O, 0.50 g (NH_4_)_2_-H-Citrate. For nitrogen limited cultures, the following composition was used (per liter): 1.00 g (NH_4_)_2_SO_4_, 1.60 g KH_2_PO_4_, 6.60 g Na_2_HPO_4_●2 H_2_O, 0.65 g Na_3_-(Citrate)_3_. The minimal medium was after sterilization supplemented with 1 mL L^−1^ trace element stock solution and 1 mL L^−1^ 1 M MgSO_4_, both sterile filtered (0.2 μm, VWR collection). The trace element stock solution contained the following components (per liter): 0.50 g CaCl_2_●2 H_2_O, 16.70 g FeCl_3_●6 H_2_O, 0.18 g ZnSO_4_●7 H_2_O, 0.16 g CuSO_4_●5 H_2_O, 0.15 g MnSO_4_●4 H_2_O, 0.18 g CoCl_2_●6 H_2_O, 20.10 g Na-EDTA. Sugar stock solutions were sterilized separately and added to the minimal medium after autoclaving. Sugar was added to the shake flask cultivations in excess, with an initial concentration of 5.00 g L^−1^ of D-glucose, D-xylose or L-arabinose, or a combination of all three in equal concentrations (1.66 g L^−1^). In the bioreactor cultivations, the sugar concentrations in the medium were 8.20 g L^−1^ D-glucose, 5.95 g L^−1^ D-xylose and 0.85 g L^−1^ L-arabinose (corresponds to the relative ratios of a lignocellulose hydrolyzate). In the continuous cultivations, the feed consisted of the finalized cultivation medium. In the fed-batch cultivations, the feed solution consisted of 100.00 g L^−1^ (NH_4_)_2_SO_4_, 206.78 g L^−1^ D-glucose, 150.04 g L^−1^ D-xylose and 21.43 g L^−1^ L-arabinose (same sugar ratio as in the starting medium). In all bioreactor cultivations, pH was kept constant at 7.0. In the continuous cultivations, as well as the non-nitrogen-limited batch cultivations, pH was titrated with NH_3_. In the nitrogen-limited cultivations, pH was titrated with NaOH.

#### Cell growth analysis

OD_600_ was measured to monitor cell growth in all cultivations. Samples were withdrawn and diluted in saline solution (0.9% NaCl w/v) to an estimated OD_600_ of 0.1-0.2 and the OD_600_ was then measured in a spectrophotometer (Genesys 20, Thermo scientific). Cell growth was also monitored through measurement of the cell dry weight (CDW). Samples of 5 mL cell culture were withdrawn in triplicates into preweighed, dry glass tubes, which were centrifuged at 2000 g in a tabletop centrifuge (CompactStar CS4, VWR) for 5 min. The pellets were washed with 5 mL saline solution and re-centrifuged at 2000 g for 5 min. The supernatant was removed and the cell pellets were dried over night at 105°C. The following day, the dry pellets were allowed to cool to ambient temperature in a desiccator, after which they were weighed.

#### Sugar and ammonia analysis, and calculation of rates

For continuous cultivations, a sample of 2 mL cell culture was withdrawn into a preweighed syringe containing 2 mL cold (~4°C) perchloric acid at a concentration of 0.13 M to stop metabolism [25]. The sample was thereafter transferred to a tube and was centrifuged at 2000 g for 10 min. 3.5 mL of the supernatant was neutralized with 75 μL saturated (~500 g L^−1^) potassium carbonate. The sample was then allowed to precipitate for 15 min on ice, after which it was centrifuged at 2000 g for 5 min. The supernatant was filtered (0.45 μm, VWR collection) and frozen at −20°C until analysis. For batch and fed-batch cultivations, a sample of 2 mL cell culture was automatically and rapidly withdrawn (<0.1 s) into a preweighed tube containing 2 mL of the same cold perchloric acid as mentioned above. Subsequent centrifugation, neutralization, centrifugation, filtration and storage steps were identical to those performed for the continuous cultivations. The sugar concentrations were determined by the use of commercially available enzymatic kits (Boehringer Mannheim Cat No. 716251, Megazyme D-Xylose Assay Kit Cat No. K-XYLOSE, Megazyme Lactose/Galactose (Rapid) Cat No. K-LACGAR). Ammonia concentrations were determined with the enzymatic kit Megazyme Ammonia Assay Kit (Rapid) Cat No. K-AMIAR. The q_S_ values were calculated as the mean over two consecutive data points, and the parameter q_S total_ accounted for the change in concentration of all three sugars.

#### 3HB and acetic acid analysis, and calculation of rates

The supernatants from the CDW samples were filtered (0.45 μm, VWR collection), stored at −20°C and used for 3HB and acetic acid analysis. The acetic acid concentration in the samples was determined with the enzymatic kit Boehringer Mannheim Cat No. 10148261035. The 3HB concentration in samples was determined with the enzymatic kit Megazyme D-3-Hydroxybutyric Acid Assay Kit Cat No. K-HDBA. This kit is specific to (*R*)-3-hydroxybutyric acid. The q_3HB_ values were calculated as the mean over three consecutive data points.

#### Gas chromatographic analysis of 3HB enantiomers

Filtered supernatants were acidified with sulfuric acid and extracted with chloroform. The extracted acids were esterified with 2-propanol and separated on a CP-ChiraSil-DEX CB column with a 40 min temperature gradient, ranging from 45 to 200°C, and detected with a FID detector.
